# [18^F^]FDG-PET/CT in adrenal lesions: diagnostic performance in different clinical settings

**DOI:** 10.1007/s12020-024-04042-5

**Published:** 2024-09-18

**Authors:** Martina Romanisio, Tommaso Daffara, Rosa Pitino, Alice Ferrero, Francesca Pizzolitto, Marco Zavattaro, Federica Biello, Alessandra Gennari, Alessandro Volpe, Gian Mauro Sacchetti, Paolo Marzullo, Gianluca Aimaretti, Flavia Prodam, Marina Caputo

**Affiliations:** 1 Endocrinology, Department of Translational Medicine, Università del Piemonte Orientale, Novara, Italy; 2 Oncology, Department of Translational Medicine, Università del Piemonte Orientale, Novara, Italy; 3 Division of Urology, Department of Translational Medicine, Università del Piemonte Orientale, Novara, Italy; 4 Unit of Nuclear Medicine, University Hospital “Maggiore Della Carità”, Corso Mazzini 18, 28100 Novara, Italy; 5 Department of Health Sciences, Università del Piemonte Orientale, Novara, Italy

**Keywords:** [18^F^]FDG-PET/CT, Adrenal, SUV ratio, Sensitivity, Specificity

## Abstract

**Purpose:**

Data regarding [18^F^]FDG-PET/CT for the characterization of adrenal lesions are limited. Most of the studies proposed the tumor-to-liver maximum standardized uptake values (SUVratio) > 1.5 as the best cut off to predict malignancy. The aim of the study was to calculate the optimum cut off in a heterogeneous population with adrenal lesions and evaluate the diagnostic performance SUVratio >1.5.

**Patients and methods:**

Retrospective analysis of adrenal lesions undergoing [18^F^]FDG-PET/CT (2013–2022) for different reasons (atypical adrenal incidentalomas, extra adrenal tumor staging). The diagnosis of benignity was assessed by: (i) histology; (ii) stability or minimal diameter increase (<20%/<5 mm) on 12-months follow-up for non-operated patients. The optimal SUVratio and performance of SUVratio >1.5 were calculated by ROC curves.

**Results:**

Forty-two consecutive lesions (diameter 36.1 ± 20.3 mm, 6 bilateral) underwent [18^F^]FDG-PET/CT (19F, age 61.2 ± 11.7 years). Twenty-nine lesions were benign, 11 malignant [8 metastases (2 bilateral) and 1 adrenocortical carcinoma (ACC)] and 2 pheochromocytomas. The SUVratio cut-off in our population was 1.55 (Sn 100%, Sp 73.7%, AUC 0.868), with similar values excluding pheochromocytomas and metastases (SUVratio cut-off 1.49, Sn 100%, Sp 96.3%, AUC 0.988). The SUVratio cut-off of 1.5 showed 100% Sn, 87% Sp, 73% PPV, and 100% NPV.

**Conclusion:**

[18^F^]FDG-PET/CT could help in decision making process avoiding unnecessary surgery. The SUVratio cut-off of 1.5 has a good performance in a heterogenous population.

## Introduction

Adrenocortical adenomas (secreting or non-secreting), adrenomedullary tumors, hyperplasia, hemorrhages, myelolipomas, cysts and malignancies (primary or secondary) could affect adrenal glands [1]. In most cases, adrenal lesions are detected on imaging not performed for suspected adrenal disease (i.e., adrenal incidentaloma). Nowadays, considering the widespread use of radiological tools, the prevalence of adrenal incidentalomas is increasing, with a frequency of 3% in the fifth decade that increases with age, and achieves up to 10% in older patients [2–8]. Regarding diagnosis, non-functional adenoma is found in 80% of cases, while the prevalence of other lesions varies ranging from 1.5–23% for pheochromocytoma, and 1.2–12% for adrenocortical carcinomas (ACC). Adrenal masses discovered during tumor stage work-up for extra-adrenal malignancies do not meet the strict definition of adrenal incidentaloma, but it is a frequent scenario and, in a series of oncological patients, 50–75% of these lesions were metastases [9].

Diagnosis of adrenal masses requires hormonal investigations and morphological characterization [10], including 1 mg overnight dexamethasone-suppression test (DST) to identify mild autonomous cortisol secretion (MACS) [11], that is characterized by high cardiometabolic comorbidities, especially in women <65 years [12].

The goal of morphological characterization is to avoid over-diagnosis and over-treatment without missing relevant diseases (i.e., adrenocortical carcinoma, pheochromocytoma). The most common radiological presentation is a small 1–4 cm unilateral homogenous adrenal mass at single phase post contrast CT scan, that does not provide a distinction between benign and malignant lesions and indicators of function. CT with delayed contrast media washout considers absolute and relative washout but data are still weak, and the accuracy of cutoffs is challenged [13, 14]. At non-contrast CT scan, lipid-rich adenomas typically show a density ≤10 Hounsfield units (HU) [15]. According to recent studies, HU cut-off < 20 is associated with a good sensitivity to predict malignancy [16] but up to 7% of metastases has HU between 10 and 20; thus, heterogeneous lesions, large masses with HU 11–20 and masses with HU > 20 require further investigations [11, 17–19]. At MRI studies, lipid-rich adenomas show loss of signal intensity on out-of-phase images. MRI is not superior to CT, and among adrenal lesions with unenhanced attenuation and high density at CT scan, about 67% of them remain indeterminate also after a chemical shift MRI [20].

Indeed, the diagnosis of adrenal lesions should not always be straightforward. Studies on radiomics (image-based texture analysis) are emerging and attracting attention for the potential role in distinguishing benign and malignant lesions even when heterogeneous, but clinical application is still limited [21]. Finally, adrenal biopsy is rarely indicated and should be performed after excluding pheochromocytoma, due to the risk of catecholamine induced hypertensive crisis and tachyarrhythmia [10].

Due to these pitfalls, in the actual clinical setting, when CT and MRI imaging are equivocal, 18F-fluorodeoxyglucose positron emission tomography computed tomography ([18^F^]FDG-PET/CT) could be proposed as a second step functional imaging [1, 10]. Several studies have evaluated [18^F^]FDG-PET/CT performance in cancer patients, finding high sensitivity and specificity in differentiating benign from malignant adrenal lesions. However, a wide range of sensitivity and specificity cut-offs has been reported [1, 22, 23]. The use of tumor-to-liver maximum standardized uptake values (SUVratio) was found to be more accurate than visual analysis, with the best threshold value that varies across studies. A prospective multicentric study calculated a SUVratio >1.5 as the best cut off to predict malignancy [sensitivity 86.7%, specificity 86.1%, positive predictive value (PPV) 56.5%, negative predictive value (NPV) 96.9%, and accuracy of 86.2%] [1, 22]. Nevertheless, some adrenal lesions could be still misdiagnosed [24–28].

Based on the limited data on [18^F^]FDG-PET/CT performance in adrenal lesions, the objective of this study was to retrospectively assess its role in the diagnosis of adrenal lesions in a heterogeneous population, calculate the optimal SUVratio in our cohort, and evaluate the performance of the SUVratio >1.5 proposed by literature.

## Patients and methods

### Patients

This was a retrospective, single-center study. We reviewed medical records of patients who received care for adrenal lesions between the 1st of January 2013 and the 31st of August 2022 in Endocrinology, Surgery and Oncology Units. The study was conducted in accordance with the Declaration of Helsinki, approved by the Local Ethical Committee (AOU “Maggiore della Carità” Novara). Informed consent was obtained by each patient.

We finally included only patients who underwent [18^F^]FDG-PET/CT imaging as a part of the management of adrenal nodules, had either a histological diagnosis or radiologic follow-up for at least 12 months. Patients underwent [18^F^]FDG-PET/CT because affected by indeterminate adrenal lesions or during staging of extra adrenal oncological diseases.

In patients with bilateral lesions, both masses were considered. Patients affected by pheochromocytomas (PCCs) and adrenal metastases were included and considered separately for the statistical analysis.

For each patient, the following data were collected: (i) demographic characteristics (sex, age at diagnosis); (ii) radiological parameters (TC/MRI) [localization (right, left, bilateral), major diameter]; iii) [18^F^]FDG-PET/CT parameters (liver SUVmax, adrenal SUVmax, SUVratio); if adrenal lesions were suspected for PCCs, a specific functional imaging [123I-metaiodobenzylguanidine (MIBG) scintigraphy or [18F]fluoro-L3,4-dihydroxyphenylalanine ([18F]DOPA PET], was performed; iv) biochemical evaluations. Biochemical parameters included: plasma cortisol after DST, 24-hour urine metanephrine and normetanephrine, ACTH (in patients with autonomous cortisol secretion), direct renin activity (DRC), aldosterone and aldosterone renin ratio (ARR) (in hypertensive or hypokalemic subjects); DHEAS, androstenedione, testosterone/estrogen and 17-OH progesterone (17OHP) (in suspicion of adrenal cortical carcinoma, or bilateral adrenal lesions).

The diagnosis of benignity was considered by: (i) histology; (ii) stability or a minimal increase in diameter (<20% and <5 mm) on the 12-month follow-up TC or MRI imaging in patients who did not undergo surgery [11].

#### Radiological evaluation

All the radiological investigations were carried out at the Institute of Diagnostic Radiology of the “Maggiore della Carità” University Hospital of Novara. All the images were re-evaluated by two independent specialists in radiodiagnostics, with specific expertise in adrenal pathology. CT scans were performed using a Philips 128-layer Ingenuity CT Elite scanner by 2 mm thickness for each scan, incrementable by 1 mm (128 kV, dose right index 17, mAs 100 and 500). The scan time was 3.3 s with a pitch of 1.49 and a rotation time of 0.4 s.

MRI mass acquisitions were performed using a Philips Ingenia 1.5 T MRI using Dixon all black T1 weighted, Dixon in and out-of-phase, TSE T2, T2-Spir and DWI sequences with adc map calculation.

#### [18^F^]FDG-PET/CT

All [18^F^]FDG-PET/CT investigations were carried out at Nuclear Medicine Unit, “Maggiore della Carità” University Hospital of Novara. An activity of 3MBq/Kg was administered and the acquisition phase after tracer injection was performed at 60 min. Images were acquired using a Biograph 16 Hirez, Siemens, PET/CT tomograph, replaced by a Philips Ingenuity-TF tomograph. Quantitative analysis was performed by drawing a region of interest (ROI) on the adrenal gland; a second ROI (diameter 3.5 cm) was drawn in the center of the liver parenchyma. SUV max was considered for both the ROI of adrenal gland and of liver. The SUVratio was calculated by dividing adrenal and liver SUVmax. A SUVratio cut-off of 1.5 was used to consider the adrenal lesions suspicious for malignancy [13].

An expert Nuclear Medicine physician reviewed [18^F^]FDG-PET/CT images, ROI and the related SUV measurements.

#### Biochemical and hormonal evaluations

All the biochemical and hormonal evaluations were performed at the Laboratory of our Hospital according to international standards.

The levels of aldosterone, renin, ACTH, cortisol, DHEA-S and androstenedione were measured by the chemiluminescence technique using the LIAISON® kits. Plasma levels of 17 -OHP were measured by ELISA technique using the DRG® kit.

For ARR calculation the limit of 3.7 was considered (aldosterone expressed in ng/dl and DRC in mU/L). Aldosterone and DRC measurements were performed after a therapy wash-out, if required.

Cortisol values <1.8 μg/dl after DST excluded MACS [10].

#### Statistical analysis

Statistical analysis was performed using IBM SPSS Statistics version 26.0 (IBM SPSS Inc). Continuous variables are expressed as means ± SD, and categorical variables are reported as numbers and percentages. Statistical significance was defined as *p* < 0.05.

A cut-off for the SUVratio was calculated by ROC curves that maximized Sn and Sp.

The sensitivity (Sn), specificity (Sp), positive and negative predictive value (PPV, NPV) were calculated for the SUVratio cut-off of 1.5 proposed by literature [1].

## Results

### Patients’ characteristics

We identified 177 patients with adrenal lesions referred to endocrinologists, surgeons and/or oncologists of our hospital. Thirty-six out of 177 patients met the inclusion criteria (61.2 ± 11.7 years), without gender difference (17 M/19 F). Fifteen patients (41.7%) had adrenal lesions on the right adrenal gland, 15 on the left adrenal gland, and 6 patients had bilateral lesions, leading to a total of 42 adrenal lesions included in the analysis.

### Radiological characteristics and diagnosis

The diameter of adrenal lesions was 36.1 ± 20.3 mm (range 14–133 mm). Twenty-three patients (63.9%) underwent unenhanced CT scan, while 7 patients (19.4%) MRI, and 5 patients (13.9%) both; one patient underwent soon [18^F^]FDG-PET/CT because of poor clinical status.

Twenty-nine lesions were classified as benign, 11 as malignant and 2 as PCCs.

Both PCCs had suggestive hormonal pattern and positive functional imaging at I123MIBG scintigraphy or [18F]DOPA PET. None of them was metastatic or recurrent.

Considering malignancies, 8 patients had adrenal metastases: 6 of them were monolateral (2 by colon cancer and 4 by lung cancer), 2 bilateral (by lung cancer) (Table 1).

**Table 1:** Clinical and pathological characteristics of adrenal lesions

Patient number	Lesion number	Surgery	Diagnosis	Age	Sex	SUVratio	Diameter (mm)	Concordance SUVratio
1	1	No	MACS	69	F	0.90	40	Yes
2	2	No	NS	66	M	0.62	14	Yes
3	**3**	Yes	NS	67	F	1.58	50	No
3	**4**	No	NS	67	F	0.67	23	Yes
4	5	Yes	NS	68	F	4.0	19	No
5	6	Yes	NS	72	M	0.89	35	Yes
6	7	Yes	NS	59	F	1.40	38	Yes
7	8	No	NS	63	M	1.26	28	Yes
8	9	No	NS	54	F	0.74	27	Yes
9	**10**	No	CS	64	F	0.95	41	Yes
9	**11**	No	CS	64	F	0.89	19	Yes
10	**12**	Yes	Aldosterone secreting adenoma	64	M	0.92	37	Yes
10	**13**	No	Hyperplasia	64	M	0.68	24	Yes
11	14	No	NS	73	M	1.38	27	Yes
12	15	No	NS	65	M	0.89	29	Yes
13	16	No	NS	48	F	1.31	43	Yes
14	17	No	NS	61	F	0.89	30	Yes
15	18	No	NS	59	M	0.69	16	Yes
16	19	Yes	Hyperplasia	82	F	4.59	133	No
17	20	No	MACS	68	M	0.84	27	Yes
18	**21**	No	MACS	77	M	0.77	31	Yes
18	**22**	No	MACS	77	M	0.77	40	Yes
19	23	Yes	NS	71	F	1.08	25	Yes
20	24	Yes	NS	63	F	1.23	35	Yes
21	25	Yes	NS	44	M	1.31	23	Yes
22	26	No	NS	61	F	0.67	36	Yes
23	27	No	NS	70	F	0.79	26	Yes
24	28	Yes	ACC	56	F	3.56	70	Yes
25	29	No	Metastases	58	F	3.63	20	Yes
26	30	No	Metastases	36	F	4.46	30	Yes
27	**31**	No	Metastases	54	M	3.59	40	Yes
27	**32**	No	Metastases	54	M	3.13	34	Yes
28	**33**	No	Metastases	77	M	1.52	19	Yes
28	**34**	No	Metastases	77	M	3.26	50	Yes
29	35	Yes	Metastases	46	M	1.58	31	Yes
30	36	Yes	Metastases	61	M	1.64	22	Yes
31	37	No	Metastases	42	F	5.24	52	Yes
32	38	Yes	Metastases	73	M	2.08	25	Yes
33	39	Yes	PCC	44	F	2.36	70	No
34	40	Yes	PCC	45	F	1.26	25	Yes
35	41	Yes	Myelolipoma	44	M	0.58	55	Yes
36	42	Yes	Malformation	79	M	0.47	57	Yes

Numbers in bold identify bilateral lesions

*ACC* adrenocortical carcinoma, *CS* cortisol secretion, *NS* non secreting adenoma, *MACS* mild autonomous cortisol secretion, *PCC* pheochromocytoma

Seventeen lesions (40.5%) were surgically treated, while 25 (59.5%) were on follow-up. When surgery was performed, the histological diagnosis was 8 cortical adenomas (7 non-functioning, 1 aldosterone secreting adenoma), 2 PCCs, 1 vascular malformation, 1 adrenal hyperplasia, 1 myelolipoma, 3 metastases, and 1 ACC (Table 1).

One patient affected by Cushing syndrome had bilateral adrenal adenomas and underwent medical treatment. The 3 patients with adenomas and MACS (in one case bilateral) presented well controlled metabolic comorbidities, thus, also considering their age, they did not undergo surgery but were in close follow-up (Table 1).

#### [18^F^]FDG-PET/CT: ROC curves

**Fig. 1:**
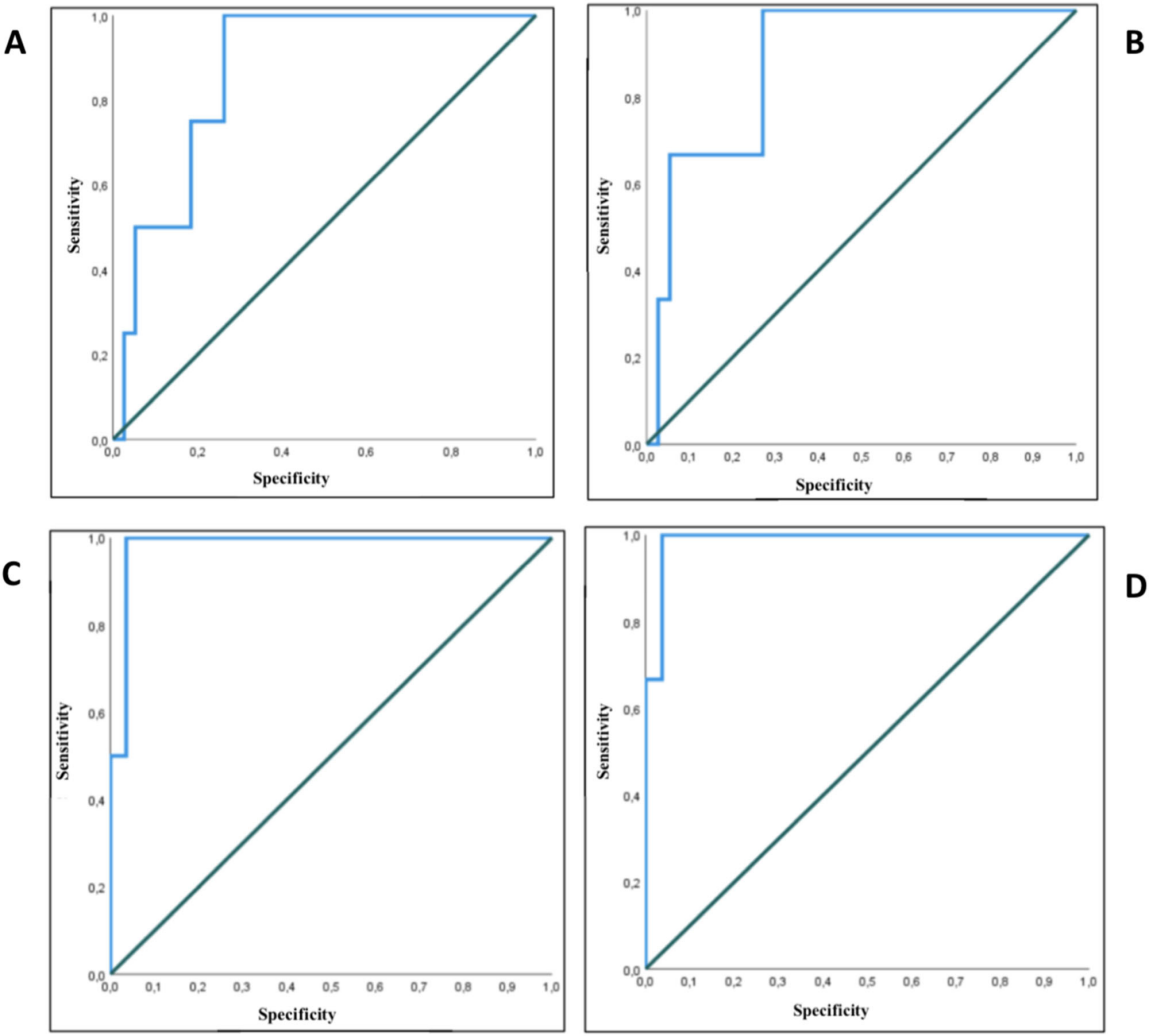
ROC curve to determine SUVratio in the entire population (**A**), excluding pheochromocytoma (**B**), excluding metastases (**C**), excluding both pheochromocytoma and metastases (**D**)

We used ROC curves to define the best SUVratio cut-off to different benign and malignant lesions in our population. In the whole population (Fig. 1A) the cut-off was 1.55 with 100% Sn, 73.7% Sp and AUC of 0.868 (95% CI 0.741–0.996). Excluding PCCs (Fig. 1B) the cut-off was 1.55 (100% Sn and 73% Sp), and AUC of 0.883 (95% CI 0.735–1). Excluding patients with metastases (Fig. 1C) the cut-off was 1.49, the Sn and Sp were 100 and 96.4% with AUC 0.982 (95% CI 0.940–1). Excluding both PCCs and metastases (Fig. 1C) we found a cut-off of 1.49 showing Sn and Sp of 100 and 96.3% and AUC 0.988 (95% CI 0.951–1).

By using the SUVratio of 1.5 [1], this cut-off agreed with the diagnosis in most of the cases (*N* = 38, 90.5%), driving correctly the management and decision-making. The 4 discordant cases (SUVratio >1.5 and non-malignant lesions at histology) were: 2 non-functioning adenomas, 1 hyperplasia, and 1 PCC. In particular, one adenoma with a SUVratio of 1.58 had a diameter of 50 mm; the other adenoma had a SUVratio of 4, a diameter of 19 mm and the mass was undetermined on CT scans (patient with history of breast cancer); PCC had a ratio of 2.36 and a diameter of 70 mm; the patient with adrenal hyperplasia had a ratio of 4.59 and a diameter of 133 mm.

The characteristics of adrenal lesions are summarized in Table 1.

The proposed literature cut-off of 1.5 for SUVratio had 100% Sn, 87% Sp, 73% PPV, and 100% NPV.

## Discussion

The recent guidelines on adrenal incidentaloma [11] provides a practical approach to adrenal masses in order to avoid overdiagnosis and overtreatment, without missing relevant diseases. Nevertheless, the clinical management of indeterminate masses is still complex. In particular, if adrenal lesion shows ambiguous characteristics on CT/MRI scans (i.e., attenuation >20 HU on basal CT, heterogeneous aspect, loss of signal intensity on out-of-phase images on MRI scans performed with the chemical shift technique), or significant dimensional growth, the use of [18^F^]FDG-PET/CT should be considered as additional imaging [1, 10, 11].

[18^F^]FDG-PET/CT is a nuclear medicine tool performed by intravenous injection of a beta plus (positron)-radiation emitting radiotracer (18-fluorine) used to label 2-deoxy-D-glucose rendering Fluoro-DeoxyGlucose ([18^F^]FDG). Both glucose and deoxyglucose are internalized via transmembrane glucose transporters and are phosphorylated, but while glucose undergoes further enzymatic breakdown, deoxyglucose remains in intracellular compartments. Since tumors show high avidity for glucose, thus taking-up more glucose and deoxyglucose than normal cells, the technique can identify cancer cells or condition with an increased metabolism, as infections. The integration between PET and CT (PET/CT) allows simultaneous acquisition of PET and CT data, adding spatial resolution information to that on metabolism. Quantitative measurement of 18F concentrations [standard uptake value (SUV)] within tissues provides the most used index. To differentiate malignant and benign adrenal lesions, the use of SUVratio has been proposed: it consists in the comparison of [18^F^]FDG uptake in the adrenal lesion between [18^F^]FDG uptake in liver. This visual comparison method has been validated for other malignant disease, i.e., lymphomas (lymphoproliferative tissue uptake compared to mediastinal blood pool and liver uptake) [29], to improve the consistency of interpretation, and the response to treatment has been standardized according to the Deauville five-point scale [30]. On the contrary, the best SUVratio to define malignant adrenal lesion has not been established yet. The best cut off value has been calculated in different studies, with high grade of variability, ranging from 0.83 to 2.5 [1, 22, 24, 31–41]. It should be underlined that analysis of the literature is hampered by suboptimal methodological study designs and test accuracies (i.e., small numbers of patients, different population characteristics, different reference standard, heterogeneity in [18^F^]FDG-PET/CT protocols and interpretation), leading to inconsistence results and lack of robustness in meta-analyses [42, 43].

Among the different proposed SUVratio cut-off [25, 44], the most methodologically rigorous and biggest study calculated a SUVratio >1.5 to predict malignancy of adrenal lesions [1]. It is a prospective multicentric study on 87 adrenal masses of indeterminate nature after unenhanced and washout CT attenuation densities. Compared to benign lesions, malignant lesions were larger in diameter, had higher HU, lower relative washout values, and higher [18^F^]FDG uptake parameters, with the optimal threshold value of the ratio for malignancy of 1.5 on the ROC curves, that showed excellent NPV (96.9%), with sensitivity of 86.7%, specificity 86.1%, positive predictive value (PPV) 56.5%, and accuracy 86.2% [1]. This cut-off has been re-evaluated in 64 non cancer patients with atypical features on CT scans [22] confirming a similar performance (sensitivity 90.0%, specificity 92.6%, PPV 69.2%, NPV 98.0%, accuracy 92.2%). Lower SUVratio cut off values were proposed by other studies [38, 41]. Tessonnier et al. [41] explored the value of [18^F^]FDG-PET/CT in the diagnosis of 41 indeterminate adrenal masses, and calculated 1.8 as the best threshold to predict malignancy, but the main limitation of the study was the low proportion of ACC. More recently, a French group studied the value of [18^F^]FDG-PET/CT in the evaluation of 66 patients with adrenal masses, included those with cancer history. Mean SUVratio values were 1.33 for adrenocortical adenoma, 4.3 for adrenocortical carcinoma and 2.68 for metastases, and the Authors proposed a cut off <1.29 to rule out malignancy [38].

Based on above, in our study, that explored the role of [18^F^]FDG-PET/CT for the evaluation of adrenal tumors presenting either as an adrenal incidentaloma or as an adrenal mass detected in oncologic patients, we demonstrated that the proposed SUVratio cut-off of 1.5 has a good performance and reproducibility. In fact, the SUVratio cut-off calculated in our population, composed of patients coming from different clinical settings (endocrinological, oncological, surgical) was 1.55. We recorded similar values excluding both pheochromocytomas and metastases by the analysis (SUVratio cut-off 1.49, Sn 100%, Sp 96.3%, AUC 0.988), nearly to the optimal cut off proposed by the previous studies [1, 22]. In all settings, the SUVratio had a good performance with an AUC ranging from 0.868 in the whole population to 0.988 excluding pheochromocytomas and metastases, suggesting that the cut-off of 1.5 proposed by other studies has a good accuracy in different populations, including malignancy.

In our population, the 1.5 cut-off agreed with the diagnosis according to the gold standard in most of the cases (38 cases; 90.5%); the 4 discordant lesions with a SUVratio >1.5 and a non-malignant diagnosis were 2 adenomas, 1 hyperplasia, and 1 pheochromocytoma. It is not surprising, since literature reports that benign adrenal lesions as adenomas [26, 45], even the small ones, and adrenal hyperplasia [25], could show a pathological uptake of glucose, although of a lesser degree, probably reflecting chronic low grade inflammation areas. Moreover, benign tumors (Weiss score 1 to 2) could harbor molecular abnormalities (i.e., 17p13 loss of heterozygosity and IGF-II overexpression [24]), highly prevalent in ACCs [46], and probably, they should be considered as pre-tumor lesions that could accumulate glucose. On the other hand, ACC and liposarcomas could exhibit low uptake values [1], while oncocytomas (even benign) exhibits highly elevated [18^F^]FDG uptake [1]. A possible explanation is that benign oncocytomas are characterized by impairment of oxidative phosphorylation processes and a compensatory excessive mitochondria biogenesis, leading to highly elevated uptake ratio values, thus representing a potential risk of false positive values.

Regarding the secreting status of the adrenal adenoma, data on glucose uptake are discordant [24, 26, 27, 45]. In particular, in the prospective study by Groussin et al. [24] on the role of [18^F^]FDG-PET/CT in the evaluation of adrenal masses (after exclusion of pheochromocytomas and patients with cancer history) no relationship between the ratio and 24-hour urinary free cortisol on univariate analysis was found; however, the Authors suggest that this finding could be related to the higher 24-hour urinary free cortisol values observed in ACC compared with adrenocortical adenomas. This topic was not evaluated in our study but could represent one of possible future goal of the work.

Currently, the main limitation of the study is the limited sample size, even if it is similar to previous studies [27, 44]. This limitation is related to the study design (single center), but, on the other hand, it ensures the use of the same PET cameras, with standardization of [18^F^]FDG-PET/CT acquisitions and reconstruction algorithms. Moreover, the real-life setting with the inclusion of a wide spectrum of adrenal diseases is a strength of the research.

In conclusion, the [18^F^]FDG-PET/CT SUVratio cutoff proposed by literature >1.5 to predict malignancy is accurate in a heterogeneous population of different clinical setting. Larger prospective studies are needed to validate these results.
